# Foster Care Dynamics and System Science: Implications for Research and Policy

**DOI:** 10.3390/ijerph14101181

**Published:** 2017-10-05

**Authors:** Fred Wulczyn, John Halloran

**Affiliations:** 1 Chapin Hall Center for Children, University of Chicago, Chicago, IL 60637, USA; fwulczyn@uchicago.edu; 2 Department of Social Work, Lewis University, Romeoville, IL 60446, USA

**Keywords:** foster care, system science, resource constraints, feedback mechanisms

## Abstract

Although system is a word frequently invoked in discussions of foster care policy and practice, there have been few if any attempts by child welfare researchers to understand the ways in which the foster care system is a system. As a consequence, insights from system science have yet to be applied in meaningful ways to the problem of making foster care systems more effective. In this study, we draw on population biology to organize a study of admissions and discharges to foster care over a 15-year period. We are interested specifically in whether resource constraints, which are conceptualized here as the number of beds, lead to a coupling of admissions and discharges within congregate care. The results, which are descriptive in nature, are consistent with theory that ties admissions and discharges together because of a resource constraint. From the data, it is clear that the underlying system exerts an important constraint on what are normally viewed as individual-level decisions. Our discussion calls on extending efforts to understand the role of system science in studies of child welfare systems, with a particular emphasis on the role of feedback as a causal influence.

## 1. Introduction

In this paper, we examine whether there is a population-level relationship, over time, between how many children enter foster care and how many children leave. Our interest in the number of children entering and leaving foster care is inspired by population biology, a field wherein core theoretical and empirical questions revolve around the size of a population over time and the resource and feedback mechanisms that influence change in that population. In these models, the carrying capacity of the host environment supports a population of a given size [1]. Birth and death processes adjust in response to the availability of resources (i.e., constrained capacity), thereby producing a population sized to fit within the capacity constraint. Resource constraints have been addressed in clinical decision-making models and organizational management [2,3]. In our work, which adopts a different empirical strategy, we use entry and exit dynamics over an extended, fine-grained temporal scale to establish whether the system behaves in a manner consistent with the presence of a carrying capacity or resource constraint.

To accomplish the empirical objective, we analogize admissions to and exits from foster care as the birth/death processes found in classical population models. Drawing from population theory, we argue that if foster care is a resource constrained system, similar to a biological population, then the behavior of the population over time should provide evidence of carrying capacity and feedback mechanisms that promote adaptive behavior within the system. Using convergent cross-mapping and other measurement strategies we examine admissions and discharges to foster care at a weekly level over a 15-year period to determine whether there is harmony in their joint ups and downs [4]. Empirically the goal is to identify parallel structure within the entry/exit dance that resonates with important policy and practice questions. If a resource constraint is present in the system, we expect that entries can be used to anticipate exits and exits can be used to anticipate entries, not at the individual level but at the aggregate or population level. In other words, the two-time series will move together in ways that we believe are both theoretically interesting and practically meaningful. Resource constraints have long been implicated in why systems do not work as well as they might [5]. Our research places some empirical findings behind the assertion and the underlying theoretical constructs.

### 1.1. Literature Review

Social work has a long and rich history of seeing the world in system terms [6,7,8]. Conceptualization of systems in child welfare has taken two separate approaches: caseloads [9,10,11] and systems reform [12,13,14]. The caseload literature, in particular, has addressed questions of resource allocation and the balance between resources and demands [9,11]. Other lines within the child welfare literature have addressed other resource questions, including funding [15], case workers [16], and case worker decision making [2,17]. These lines of research focus either on the micro context of clinical practice, or on the macro context of policy, with the analysis centered on the aggregate or annual level.

The in and out flow of children through the foster care system also evokes similarities with system dynamics, organizational change, and supply chain management. The systems dynamics approach is a modelling-forward attempt to reconstruct the behavior of an interacting, interrelated, dynamic system rooted in control systems theory [18,19]. Similarly, management literature focused on organizational change focuses on organizations as complex systems, seeing multiple barriers and opportunities for change behavior [20,21]. Supply chain management has applications in many different fields—from manufacturing to health care—focusing on the adaption of systems to fluctuations in inputs and outputs. In health sciences, supply chain analysis has been used to study hospital admissions by looking at how physicians were incentivized for certain procedures and how those incentive structures create emergent professional behavior [21].

With regard to the extant literature, the empirical strategy taken in the paper builds on this literature but represents a significant departure from how placement in foster care has been studied in the past. Typically, the decision to place children in foster care is framed as a clinical decision informed by child, family, and contextual factors. Decision-making models consider the influence these factors have on whether a child is placed into out-of-home care [22,23]. Typically, the dependent variable in a standard general linear model is the log odds of placement, a simple binary. In the case of exits from care, the dependent variable most often is the unit time probability of leaving foster care. Our approach, similar to that of a population biologist, focuses instead on aggregate population behavior. In our models, the quantity of interest is the number of children admitted and the number discharged during an interval of time. Our specific interest is in how the quantity changes over time at a small temporal scale (i.e., weeks). Trend data are routinely produced by foster care agencies. We contend, however, that these data, which tend to be annualized, remove operationally useful information from conversations about how systems work and why we see the population changes we see. We want to add that lost information back into policy and practice discourse.

### 1.2. Theoretical Constructs

We are interested in the role of resource constraints on the number of entries and exits to foster care. Resource constraints in this model are aligned with the idea of a carrying capacity. The carrying capacity represents the resources available to support a population that depends on those resources. There are many types of resources supporting the foster care system. Funding is usually the first constrained resource that comes to mind, but we do not focus on funding levels. Rather, we are interested in the types of resources money can buy. Prominent among those resources, the number of beds and worker time are two that have attracted most of our interest [5,24].

In this paper, we are focused on the number of beds as a capacity constraint that affects the number of children entering and leaving out-of-home care. We do not directly measure the number of beds. Instead, we observe whether patterns in the entry/exit time series data are consistent with time series data emanating from systems wherein there is a known resource constraint [1,5]. If the results match, then we have one piece of evidence as to the presence of a resource constraint.

We focus the study on congregate care, defined to include group and residential care. The choice of congregate care reflects our interest in studying resource constraints in a sector of the system characterized by the fixed cost structure of the underlying resources. Congregate care beds are expensive, relatively inelastic in the face of short-term fluctuations in demand, and notoriously difficult to add and/or subtract, although for different reasons. Because beds are relatively hard to add, the decision to subtract beds in the face of unknown demand for the service is a difficult one for managers and policy-makers alike. If a resource constraint ties entries and exits together, we believe the empirical signature of the constraint will be easier to see within the entry/exit time series generated by children moving in and out of congregate care.

### 1.3. Motivation

Our study is motivated by an interest in knowing whether the foster care system belongs to the class of systems referred to as complex, adaptive, or non-linear, depending on how the words are defined and used in combination [25]. Observers of the foster care system use the term *system* frequently when talking about what path system reform should take, but the truth is the empirical justification for using those terms has yet to be firmly established. More pointedly, complex or adaptive systems ought to exhibit behavior consistent with theoretical expectations. If the system is complex and adaptive then the way we think about manipulating the system in favor of the children and families it serves is presumably enhanced. Without that evidence, our approach to system reform should probably omit thinking that depends on adaptive, non-linear causal models [26]. Suffice it to say establishing the adaptive qualities of a system requires a different set of statistical tools from those used to understand placement experiences [5]. Our aim here is providing a descriptive understanding of what one finds when the tools of adaptive, non-linear systems theory are applied to the foster care system generally and the congregate care system specifically.

## 2. Materials and Methods

The data for this study are very simple. From the administrative records maintained by a single, medium-sized U.S. state (i.e., big data in this context), we calculate the number children admitted to and discharged from care by care type each day for fifteen years. The counts involve children between the ages of 0 and 17 at the time of admission. The underlying data set consists of two-such time series with the two counts arrayed in their temporal order. The first consists of 5475 days of admissions, which is equivalent to 782 weeks, 182 months, and 60 quarters of three-month duration. A replicate time series is also available for the discharges. Together the two times series allow us to say how many children were in care at the start of the day, how many children were admitted during the day, how many children were discharged during the day, and how many children were therefor in care at the end of the day (i.e., the starting value for the next day). This is a standard dynamic population model consisting of stocks and flows and a time step of one day. Over the study period, from 1 January 2000 to 31 December 2015, there were 24,449 admissions into congregate care and 25,188 discharges from congregate care. It is important to note that there is relatively little measurement error in these count data when compared to studies that use natural populations.

To be more specific about the counts going into the time series, the admissions counted on a given day are based on the start date of any placement into congregate care, which is defined as group or residential care. At the child level, a congregate care placement may have been the child’s first time in congregate care or the n^th^ congregate care placement. The only observable of interest is the start date of the placement. All placements with that start date were counted as admissions on that date. The parallel process was followed for discharges. The number of exits represents the number of congregate care placements that ended on that day. Although it can happen, children are usually not admitted and discharged on the same day. When it does happen, the admission and exit are counted. We note this possibility to make the point that the admissions and discharges on a given day are rarely coupled at the child level.

When children move from one congregate care placement to another in succession, the entry and exit dates refer to the start date of the first congregate care placement and the end date of the last in the series. We refer to consecutive placements in the same care type (not necessarily the same facility) as care type spells; the spell represents a continuous period of time in the specified care type. Though we do not address other spell types in this paper, the list of care type spells includes foster care and relative care. Finer distinctions are possible. For an exploratory study of this nature we opted to focus on congregate care as a more general care type spell. If the results justify a deeper look, the time series data can be adapted to accommodate more refined measures of entry and exit volume and care type. For example, we are able to differentiate between group care and residential care. We are also able to disaggregate care type spells by the agency the supervises the home. These finer distinctions will be examined in subsequent papers.

### 2.1. Research Questions and Hypothesis

Theory predicts that when resource constraints are present in a foster care system, the admission and discharge time series will move together in ways that are a representation of the resource constraint [1,2,5]. Depending on the system, co-evolving admission and discharge time series data may be prompted by causal mechanisms that allow one to anticipate exits from entries and entries from exits. Our first questions, covered in this paper, focus on whether the time series data provide evidence for system effects consistent with presence of a resource constraint or the carrying capacity of the system.

The first question is descriptive. What does the weekly time series of entries and exits over fifteen years look like? Is there, without the aid of statistical manipulation, a readily observable structure to the data? Structure in this case refers to patterns in the relationship between time and the volume of entries and exits that invoke a policy or practice relevant narrative. For example, one might expect to see higher volumes at certain times of the year (i.e., seasonality) because of a natural ebb and flow tied to calendar cycles. There may be other visible structures; the descriptive narrative will point out the most obvious.

The second question foreshadows the answer to the first. Theoretically interesting, empirically verifiable patterns are difficult to see in count data from 782 weeks. The time scale is small and the extent of variability from one week to the next is considerable. Patterns are hard to see, especially if we are interested in whether the two time series evolve in a causal response to each other. Thus, the subsequent questions are built around conventional tools used to observe otherwise hidden structure in two related times series. For example, we start by asking: what is the correlation between entries and exits. We use a linear model to observe the co-variance within the two data sets. Embedded within the question about the linear relationship is a question about time—how much time is needed to observe a change in admissions (i.e., a stimulus) and a change in discharges (i.e., the response). Knowledge about the state of the system takes time to pass through the system. Known as the adjustment parameter in linear partial adjustment models [1,5], theory maintains that it will take some time before an admission echo is observed in the discharges and vice versa. In classical population models, this is known as the lag. The lag length is also linked to the timescale at which adaptive mechanisms are at work within the system. When properly set, the lag provides the right viewing space for seeing structure in the data. Our second question, therefore, focuses on whether lags of different lengths reveal structure in data.

The third question probes whether the entry-exit time series data map onto one another. As in most quantitative approaches, the central question is whether a pattern of entries produces an echo in the exit data some time later, with how much later defined by the lag. That is, at a given lag, the likelihood of seeing an echo is greater than at other lags. We use the lag to define a specific set of entry/exit pairs to understand whether there is a co-evolving structure. Many such comparisons are boot-strapped and evaluated for their similarities. The difference between this analysis and the linear analysis described in the second step is tied to the distinction between correlational analysis and causal analysis. Question 2 provides evidence of correlation. Convergent cross-mapping, which is the method used here, has been used to characterize causal relationships within cross-mapped time series data sourced from the same system. Although we do not assert a causal relationship, we do use the results to argue for a deeper look at the problem.

### 2.2. Linear Analysis and Lag Determination

In our model, we are naturally interested in the order of the paired counts. If there is a relationship, we would expect it to happen through time. Some systems respond to feedback quickly, some more slowly [1]. For that reason, the methods used in the study are explicitly cognizant of temporal sequence. We are still interested in ordered pairs, but the pairs are more carefully assembled in this part of the analysis. We select the admission count on a given day and pair it with discharges some number of days later. The lag is how far forward or backward one needs to look to find a link between admissions and discharges or vice versa. To determine lags, we performed autocorrelation analysis of the dependent variable across a limited time horizon—here we selected ten weeks.

In first analysis, we looked for linear patterns in the number of admissions across time. After the lag lengths with the highest autocorrelation factors are determined, we plot those potential lags on three axes and evaluate the density of those plots as compared to similarly plotted random time series. Together these analyses help establish whether there is structure within the raw time series. The meaning of those structures is where utility for policy and practice enters into discussion.

### 2.3. Convergent Cross-Mapping

Convergent cross-mapping (CCM) is a method rooted in the idea that certain classes of system produce time series data with a known mathematical form. The convergent cross map and related techniques are used to look for those structures. If the structure is present in the time series then we then have evidence that the observed data come from a system that can be described using the underlying functional form. For example, linear systems produce time series data of a very specific form. If the data represent admissions and there is a linear relationship with time, the number of admissions will grow or decline in linear fashion. We can say then that with the passage of time, the number of admissions will grow. Of course, if there is a limit on growth, the linear model will be less relevant. More complex systems, non-linear systems as an example, will produce a more complicated time series, but the absence of visual structure may not be a fair representation of the underlying system. For these types of time series data, more sensitive measurements are needed to see the underlying structure and the accompanying narrative.

Developed by theoretical ecologist George Sugihara and others [4], CCM looks for causal relationships in the time series data emanating from the same system. Fundamentally, over time, the time series will evolve in a manner that reveals clues about the underlying system. Said another way, if no such structure exists—e.g., the points are random—then the likelihood a coherent system is responsible for why admissions (or discharges) change from one week to the next is much less likely. CCM is one tool then for distinguishing between random and non-random time series.

In our study, we hypothesize that the behavior of the system is governed, in part, by the resource dependencies which systematically impact the number of admissions and discharges. CCM tests for the relationship between coupled variables by building an increasingly large library of randomly drawn pairs (starting with a small number of pairs and proceeding to a large number of pairs), predicting that as library lengths increase so too will the strength of the relationship between the two variables. CCM, moreover, is able to analyze this coupling in higher dimensional space, allowing us to gain insight into the nonlinear behavior of time series. A measure of this dimensionality—embedding dimension—is reported along with the CCM coefficient, where the value of zero is a linear system and higher dimensions indicate more complex dynamics that link admissions to discharges.

## 3. Results

### 3.1. Admissions and Discharges through Time

**Figure 1:**
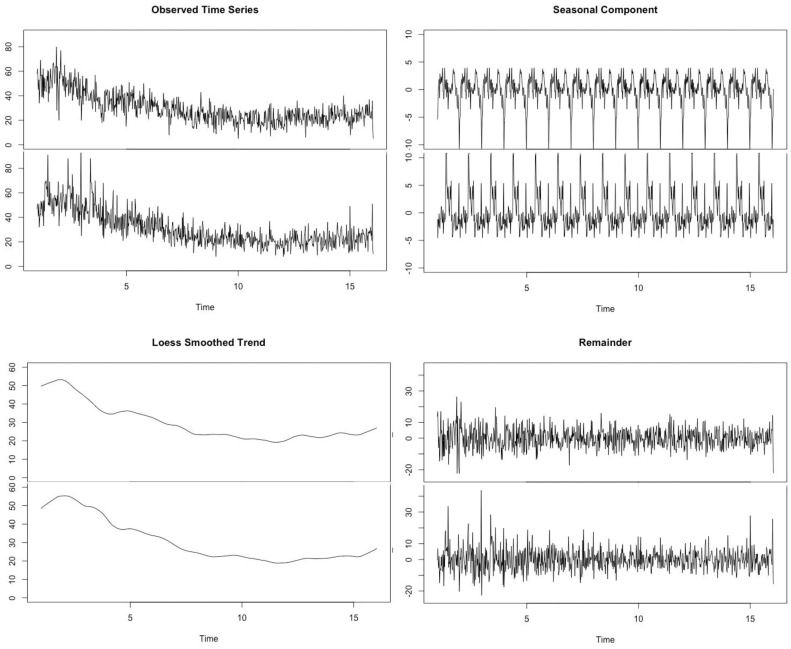
Weekly congregate care admissions (upper panels) and discharges (lower panels) in four components. Upper left—The observed raw admission (top) and discharge (bottom) data. Upper right—The seasonal patterning of those series in an annual period, with admissions on the top and discharges on the bottom. Lower left—The loess smoothed trend series after extracting the seasonal component. Lower right—The unexplained remaining variance in the observed series after considering the seasonal and trend components.

Presented in Figure 1 are four views of the weekly admission/discharge time series data: (1) a raw time series plot, where the entries or exits are plotted against time. In this plot (and in plot 3) the y-axis represents the aggregate number of placements each week; (2) a seasonal plot which evaluates the annual periodic fluctuations found within the time series; (3) a trend plot which smooths the raw time series plot using the periodic seasonal plot; and (4) a remainder plot which evaluates variance between the smoothed plot and the raw data.

The first panel shows the raw data. Both admission and discharge time series show a decline in number. The discharge counts are somewhat behind the admission decline because of the time lag. The number of discharges will fall in response to the falling number of admissions at a lag roughly proportional to the average time in care. Our analysis generally seeks to understand the relationship, if any, between the admission/discharge cycles seen in these two plots.

Apart from the general decline, more specific patterns are difficult to see. There may be structure in these data—structure that aligns with an explanatory narrative about how the system works—but that structure and the resulting narrative are hard to extract with the naked eye. We contend the structure is present but special analytical approaches are needed to reveal that structure.

The second panel shows seasonal decomposition plots for the two time series. One element of the narrative regarding the volume of admissions and discharges is their seasonal nature, a pattern that is revealed in the seasonal plots. These data point to evidence of low admission activity in December and January of each year and somewhat elevated admission activity in April and May and then again in September and October. The valleys of the admissions time series tend to have a larger amplitude than the peaks. The discharges tend to find relatively higher seasonal amplitude in the peaks than the admissions, though the peak and valley patterns are similar to those in the admissions time series: high exits in May and June and low exits in November and December.

The lower left panel shows the smoothed admission and discharge plots. The loess smoothing across quarterly periods was done in conjunction with the second and first plots. The trend plot is closer to what policy makers, providers and advocates look at when assessing the state of the system. Congregate care admissions (and exits) have trended downward with a slight uptick at the end of the time series. As states are trying to reduce the use of congregate care, the narrative that goes with a decline is a generally positive one. The lower right panel considers the difference between the observed time series and smoothed trend. The seasonal and smoothed trend plots prompt a narrative about the state of the system because of the structure visible in the two-dimensional profile (i.e., volume plotted against time); the question is whether there is any structure in the remainder plot. We contend that the structure in the remainder plot says as much if not more about the underlying system than either the second or third panel, provided that structure is detected.

### 3.2. Linear Analysis and Lag Determination

What follows are several steps taken to characterize the otherwise hidden structure in the data. Using the panel one data, which includes the data in the remainder plot, the first step we took was to see if there were any linear relationships in the data across time. Table 1 reports the autocorrelation analysis with the coefficient of correlation (rho) reported for the time series data. To establish the pairs used for the standard correlation, different lag lengths are used. An entry for one week is paired with an entry from another week a fixed number of weeks in the future. The correlation presented is the correlation between the two columns of data.

For congregate care, the autocorrelation for a lag of one week generated the highest value; for the admissions, a lag of two weeks had the highest autocorrelation. These lag times were confirmed by simple bivariate linear regression models. For admissions, a lag of two weeks explained a slightly higher amount of variance than the lag of one week. For example, in the congregate care series, we obtained an *R*^2^ of 0.2402 for a lag of one week versus a *R*^2^ of 0.2499 for a lag of two weeks.

**Figure 2:**
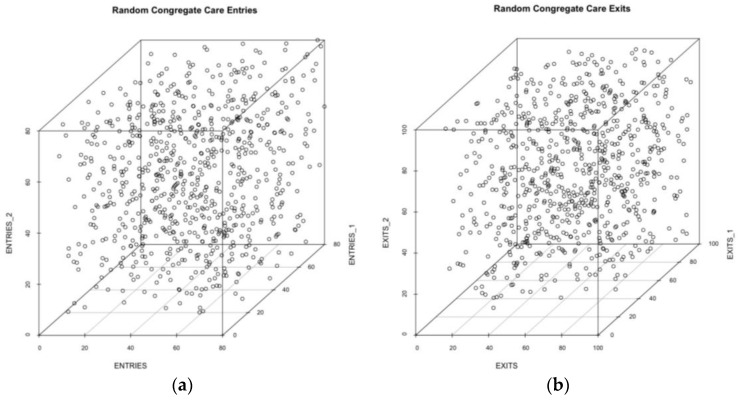
Three-dimensional lag plot of the variable of interest (x-axis) compared to the variable position in the first-order lag (y-axis) and second-order lag (z-axis). In the entries plot (**a**) and the exits plot (**b**) the data is randomly generated using the observed parametric bounds of the time series.

Another way to examine structure within the times series is to project the data into a three-dimensional state space. Random time series will seemingly fill the space evenly; more structured data will form a cloud with structure. To illustrate the point, we start with three-dimensional time series plots using random data. The random data are generated from a time series bound by the values found in panel 1 above. The points represent the number of admissions (or discharges) to congregate care at time 0, (x), time *t* – 1, (y), and time *t* – 2, (z), with the lags in weeks preceding time *t*. The results are in Figure 2.

As expected, the random plot shows no structure, which means that the next value for admissions in the time series is as likely as any other value, provided it falls within the range of values ever produced. Among other things, the random time series means that there is no apparent force within the system compelling or constraining the number of admissions or discharges in some way.

**Figure 3:**
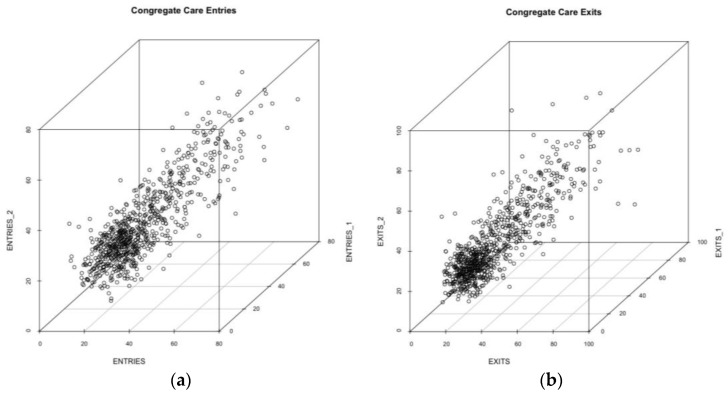
Three-dimensional lag plot of the observed entries (**a**) and exits (**b**) to congregate Care, 2000 to 2015, in their state at time zero (x-axis) compared to the variable position in the first-order lag (y-axis) and second-order lag (z-axis).

In Figure 3, we show three-dimensional scatter plots for observed admissions and discharges to congregate care. As hypothesized, the data for the congregate care series are more tightly patterned than the random time series. This means that the next point is more likely to fall within a specific region of the three-dimensional space. The tighter clustering suggests that system structure might relate to “memory” in the system of immediately preceding system states, that is that there may be linkage between present observed values within the system and prior entry/exit patterning. Some evidence of that structuring or linkage reinforces our question about feedback-governed patterning in congregate care systems. The non-random nature of the plot is, we believe, a marker for structures within the data that have explanatory power pertaining to the system that produces the time series data.

### 3.3. Cross-Mapping

To push forward with this assertion, we used CCM to assess whether the underlying system is likely a constrained one. That is, admissions and discharges move together because they are linked to the carrying capacity of the system. Why might we observe such a constraint? Congregate care is a fixed cost asset. Each bed within a facility has a cost associated with it. When a bed is occupied, the organization that manages the facility has the revenue to cover the cost of that bed, but only at the margin. The utilization rate represents how many days out of a fiscal year all the beds (i.e., bed days) are filled. The target utilization rate is the utilization rate needed by a provider to achieve a healthy financial equilibrium, assuming there is a revenue target aligned with bed utilization, which tends to be the case. The utilization level or target, in an adaptive system, is thought to motivate behavior throughout the system. The evidence we present assesses whether the behavior of the time series data is consistent with constraints built up around the link between revenue, bed utilization, and bed supply. The reason admissions do not take just any value is because admissions are constrained by the discharges and vice versa because of a utilization target. If we find a link between admissions and discharges, then we have evidence of what might best be described as a supply effect on admission and discharge dynamics. Conditions that favor admissions exist when there have been discharges; conditions that favor subsequent discharges exist when admissions are forthcoming.

In the entries-favor-exits analysis for congregate care shown in Table 2, we found a CCM coefficient of 0.7660 (*p* < 0.001) and for the exits-favor-entries data a coefficient of 0.7979 (*p* < 0.001). CCM coefficient values range from a completely deterministic system (1.0) to a completely random system (0.0). In this system, we see high values for the CCM coefficients, which supports our hypotheses that congregate care is a resource constrained system, where the signals for such constraint can be detected by time series analysis. This lends evidence to the hypothesis that a coupled entry-exit structure exists within the congregate care system. In the exits-favor-entries time series, the CCM coefficient is high in comparison to the bivariate regression and correlation coefficients for the same data. In the entries-favor-exists time series, though the CCM coefficient was high, it was in a similar range as the bivariate regression coefficient. This suggests that there are dynamics in both the linear and nonlinear space for the entries-favor-exits subsystem.

## 4. Discussion

Policy-makers, practitioners, and researchers all refer at one time or another to the foster care system and their desire to improve the *system*. Undoubtedly the reference refers to the parts of the system and their interconnectedness. However, missing from the policy, practice, and research discourse is a concerted effort to understand specifically what the word “system” means in the foster care context [26]. Systems have been studied in fields as diverse as physics, chemistry, and biology [27,28]. Similarly, social service management literature has focused on the impact of resource constraints at the street-level [29,30,31]. Unfortunately, little of that thinking has penetrated foster care systems research and how system insights can be used to better manage the system. The later point is, of course, limited by what we find when we hold the foster care system up to a theoretically and empirically motivated caricature of what a foster care system is. Is the foster care system linear or non-linear? Is the foster care system a complex, adaptive system? Additional answers to these questions will likely have an impact on how we approach improving the system [32,33].

Because there is a paucity of empirical research that explores these ideas in the foster care context, we set out to answer a series of questions motivated by population biology. Among the disciplines looking at systems, biologists utilize several models that align well with the principal questions facing policy-makers, practitioners, and advocates: why is the foster care population as large as it is. Fundamentally, the foster care population is a by-product of admissions and discharges just as a natural population is a function births and deaths. From that starting point, we looked to see whether population models have relevance to the foster care problem. In particular, we were interested in theories that connect resource constraints (i.e., carrying capacity) and the evolving admission/discharge time series.

Although only descriptive, the results are consistent with the theoretical expectations that predict adaptions in population dynamics in the face of resource constraints. When compared with a random walk of points constructed from the same set of potential values, the plot of points that characterize the observed admissions and discharges in their temporal order are clearly constrained. The next point in the time series is unlikely to fall just anywhere in the state space; rather there is an unobserved constraint that affects where the next point (i.e., the count of admissions or discharges) will fall. The question then is whether the constraint in place has theoretical interest and practical utility.

To push that idea, we looked for a pattern in the admission/discharge data that suggests a constraint organized around resources or the system’s carrying capacity. Such a capacity constraint would, theoretically, compel changes through feedback in admissions to offset discharges and vice versa. The evidence for a capacity constraint that ties admissions and discharges together is noteworthy if not incontrovertible. The admissions-cause-discharges and the discharges-cause-admissions dynamic, as revealed in the time series data is strong, even through times when the size of the system (i.e., the number of children in congregate care) is shrinking. Congregate care beds are inelastic with regard to demand over the near-term. At a population level, admissions can only happen if a bed is available. Discharges generate available space; children waiting admission depend on a discharge. These dynamics are not per se observable at the individual level. Instead they are captured in the adaptive admission and discharge flows that determine the size of the foster care population given the carrying capacity.

If the evidence is consistent with the presence of a capacity constraint, the evidence does not speak to how the adaptive mechanisms manifest themselves. In the end, the decision to admit or discharge a child is made by people. If systems are adaptive, what role do the micro-level actions of these individuals play? We contend that this is a central problem for social scientists hoping to understand how systems work, their influence on individual actors within the system and vice versa. Our findings point to the powerful influence macro-forces play within the decision-making context. Much like gravity constrains the choices we make about how to move around, the macro forces at work may have a presence, though rarely measured explicitly, that shape the range of options considered by decision makers. If so, theory suggests that managing carrying capacity in alignment with demand is one key to controlling the effect of constraints on clinical decision-making.

## 5. Conclusions

The findings offered here begin to characterize the true nature of the foster care system. Specifically, the evidence suggests that the foster care system likely belongs to the class of systems recognized as complex, and adaptive. In systems of this type, feedback plays an important part in the causal mechanisms that regulate how the system evolves over time [34]. Feedback, in this case, is different from the narrative managers use to monitor and report on how well the system is working, as in the type of verbal feedback offered as part of quality improvement cycles [3]. Feedback in dynamical systems refers to the complex, bi-directional causal mechanism that exists between parts of the system and compels adaptation over time. Within systems theory, feedback is a ubiquitous feature invoked when trying to understand how the system works and how one might more efficiently control the system. This produces a management narrative that focuses on whole populations—systems—in a dynamic process of adaption and adjustment rather than on individual case management [33,35]. Simply by conceiving of foster care systems as a holistic, interdependent, dynamic system of feedback and response as opposed to a series of independent clinical decisions, a new series of policy and management intervention points are revealed [33]. From our perspective, a deeper understanding of systems principles will strengthen policies and other tools used to manage the child welfare system on behalf of children and families.

## Figures and Tables

**Table 1:** Autocorrelation of lagged time series variables.

Lag Lengths	CC Entries	CC Exits
1 week	0.6993	0.7232
2 weeks	0.7121	0.7163
3 weeks	0.6765	0.7035
4 weeks	0.6773	0.7187

**Table 2:** CCM coefficients in congregate care entry and exit time series.

Outcome	Predictor	Regression Beta	Correlation Coefficient	CCM Coefficient
CC Entries	CC Exits	0.5607	0.6533	0.7979
CC Exits	CC Entries	0.7720	0.6898	0.7660
